# Report of Mr. Guthrie's Practice in Diseases of the Eye

**Published:** 1829-01-01

**Authors:** 


XLIII.

### ROYAL WESTMINSTER OPHTHALMIC HOSPITAL.

In our last number we gave a report of a clinical lecture delivered by Mr. Guthrie to the students of the hospital, on the use of several remedies which had been introduced by him, and used on different principles from those usually entertained. The result has, in many instances, been something resembling magic—cures having been effected in cases in a very short time, which cases long resisted all previous treatment. In acute diseases, the success had not been less; and it is our intention, from time to time, to report the treatment of a certain number of cases of each kind of disease, so as to bring before the public the practice of this hospital in every species of complaint to which the eye is usually liable. We may premise with advantage, that the patients, often amounting to 160 and upwards, have tickets given them on their arrival, and are called up in succession, a proceeding which prevents the crowding and crushing which took place before it was adopted. Each case is given in charge to a particular student, whose name is at the bottom of the letter, and on whose diligence and attention the statement of it depends; Mr. Guthrie merely superintending the treatment, as his intervention may seem to be necessary, or is required by the student.

*Case* 1. Thomas Levignon, aged 18, admitted September 23d, 1828, having been ill one month with an affection of his eye, apparently catarrhal inflammation, which had nearly deprived him of sight, when he received a blow from a pot, (on the 19th,) which gave him great pain, and augmented the swelling. He applied alum-water to it, which caused it to subside. At present the conjunctiva is inflamed, the cornea muddy, the discharge viscid, tears hot, pain extends to the side of the face and teeth, and cannot sleep at night on account of it. Mr. Guthrie said this was a case which appeared to require, in almost an imperative manner, the abstraction of blood; but he was disposed to give the students an opportunity of seeing the effect of a different mode of treatment, and applied the ung. argent. nitrat. directing the patient to attend next morning.

24th. Says he is much better, and greatly relieved from pain. The treatment was severe, but it did him good, and he wished it to be repeated.

26th. Rep. ung. 30th. Rep. ung. October 2d. Rep.

October 14th. Ointment repeated every second and third day. Patient discharged cured. Case kept by Mr. Taylor.

*Case* 2. William Saunders, aged 35, admitted with chronic inflammation of both eyes, of three months' standing. Ordered the ung. argent. nitrat. to the left eye only on the 7th October.

9th. The left eye, to which the ointment has been applied, is better.

11th. The ointment to be applied to both. Pulv. jalap. c. ℈j.

14th, 16th, 18th, 22d, 23d, 25th, 28th, 30th. Ung. argent. nitrat. pulv. jalap. c. ℈j. Nov. 11th. Discharged cured. Case kept by Mr. Griffin.

*Case* 3. Joseph Caries, aged 25, admitted October 18th, 1828. Was attacked five months ago by inflammation of the left eye, in consequence of having got cold; complained of pain in it, attended with itching, and accompanied by a copious flow of tears, which considerably increased the pain. When he applied here for relief the symptoms were these. The eye suffused—chronic inflammation of the cornea and sclerotica, with opacity of the former—the iris slightly irregular—slight tarsal inflammation. Ung. argent. nit. No constitutional treatment.

20th. Repeat. 23d. Has obtained considerable relief. 25th. Repeat. 28th. Much better—sees now pretty well, which he could not do when he first attended—inflammation nearly gone. 30th, 31st, Nov. 4th. Repeat the ointment. Nov. 19th. Discharged cured. Case kept by Mr. Molineaux.

*Case* 4. Louisa Todd, eight years old, admitted October 16th, having been ill four days. Numerous red vessels run on the conjunctivæ of both eyes and eyelids—blush of redness on the sclerotica—slight chemosis of the right eye—great lachrymation, with discharge of yellowish matter—intolerance of light, and considerable pain in both eyes. Tongue clean. Ung. argent. nitrat. to both eyes. Pulv. jalap, c. ℈ss. 18th. Eyes greatly improved. Repeat ointment and powders. 25th. Better, and repeat. 28th. Improving. 30th. Repeat.

Nov. 11th. The ointment and powders have been repeated every second or third day. All appearance of inflammation is gone, and she says she is well. Discharged. Case kept by Mr. Nicol.

*Case* 5. Ann Allen, æt. 23, 14th Oct. 1828, admitted. Catarrhal inflammation very bad. Ung. argent. nitrat. Cured by one application. Case by D. Griffin.

*Case* 6. Thomas Cooper admitted Nov. 4th, 1828, with inflamed conjunctiva, and commencing chemosis around the cornea —intolerance of light, and feeling as if particles of sand were within the lids—increased flow of tears, with mucous discharge—left eye much worse than the right. Taken ill the 1st. The ung. argent. nitrat. to both eyes.

Nov. 6th. Much better. 8th. Gradually improving. 11th. Much better. 13th. Cured. Case kept by Mr. Griffin.

This boy, the son of a pork butcher at Lisson Grove, caught the complaint apparently from his younger sister, a child who had been brought to the hospital with purulent ophthalmia, both upper eye-lids being complely everted, so as to make her appearance perfectly hideous. The cornea of the left eye was sloughing —chemosis of the right—great purulent discharge. She was cupped to eight ounces on the right temple—the ung. argent. nitrat. was applied every second day, and the lotio aluminis on the intermediate one. This child is also well, the right eye being saved—the left, which was sloughing when she was admitted, having been lost.

*Case* 7. Mary Rank, æt. 22, admitted Nov. 13. Sclerotic inflammation. About eight days ago was attacked with violent pain in the eye-ball and temple. It is always worse in the morning—light increases the pain—the vessels are of a pale red, as seen running in the sclerotica. She was purged, bled from the arm, and had leeches applied without any relief. To lose blood by cupping to 20 ounces from the left temple. Hyd. submur. gr. ij. ex. coloc. c. gr. ij. statim. Magnes. sulph. ℥ss. vespere. 14th. Eye quite free from pain; head still a little painful. Pulv. jalap, c. ʒiss. 15th. Cured--returned thanks. Case by Mr. Dyce Guthrie.

